# Volatile Compounds Governed by Single Recessive Gene Impart Aroma in Sponge Gourd (*Luffa cylindrica* L. Roem)

**DOI:** 10.3390/plants11212881

**Published:** 2022-10-28

**Authors:** Tribhuvan Chaubey, Vidya Sagar, Ramesh Kumar Singh, Chandan Singh Chanotiya, Sudhakar Pandey, Prabhakar M. Singh, Pradip Karmakar, Jagdish Singh, Bijendra Singh, Dhananjay Pratap Singh, Koshlendra Kumar Pandey, Tusar Kanti Behera

**Affiliations:** 1 ICAR-Indian Institute of Vegetable Research (IIVR), Varanasi 221305, India; 2 CSIR-Central Institute for Medicinal and Aromatic Plants, Lucknow 226015, India; 3 Acharya Narendra Deva University of Agriculture & Technology, Kumarganj, Ayodhya 224229, India

**Keywords:** sponge gourd, basmati-like aroma, volatile compounds, hexanal, fruit colour, inheritance study

## Abstract

As a vegetable crop, sponge gourd is widely consumed worldwide due to its health promoting and nutraceutical value. This study describes genetics of an aromatic genotype VRSG-7-17 and deciphers the genetic control and volatile compound composition of sponge gourd. To study the inheritance of this trait, a cross was made between aromatic light-green-fruited VRSG-7-17 and non-aromatic dark-green-fruited VRSG-194 genotypes. The F_1_s were found to be non-aromatic and have a green fruit colour. Chi-square (χ^2^) analysis of backcross and F_2_ population segregating for aroma suggested that the inheritance of aroma in VRSG-7-17 is governed by a single recessive gene in a simple Mendelian fashion. The SPME–GC/MS analysis of the volatile compounds suggested that the compounds responsible for Basmati rice-like aroma were mainly hexanal, 1-octen-3-ol, 3-octanone and limonene. The aroma persists in the cooked VRSG-7-17 fruits, that did not lose fragrance traits at high temperatures. The inheritance of fruit colour was found to be controlled by a single gene with incomplete dominance. The segregation analysis showed that the aroma and fruit colour were not linked, and they segregated independently. The findings will lead to understanding the inheritance of the aromatic compounds in the sponge gourd and may be utilised in the breeding programmes for developing improved aromatic varieties.

## 1. Introduction

The flavour and aroma in food are determined by the chemical senses of taste and smell. The odour receptors of the organs responsible for smell detect aroma in food. This is because the foods contain aromatic compounds such as alcohols, aldehydes, amines, esters, ketones, lactones and terpenes that have multiple functions [1]. India is home to a number of traditional aromatic foods, of which Basmati rice is the most popular. The word “Basmati” appears to be derived from two Sanskrit words, *vaas* (fragrance) and *matup* (possessing), implying that the one which possesses fragrance is called Basmati [2]. A harmonious aroma blend in the food increases its acceptability and likeability. For example, aromatic rice has fetched a higher market price than non-aromatic rice and has remained in high demand in local and global markets. Apart from basmati rice, the aroma has been reported as one of the quality characteristics of a variety of crops, fungi, microbes and processed foods [1]. Likewise, consumers’ preference for aromatic sponge gourd varieties over non-aromatic varieties has resulted in the high price of aromatic cultivars [3].

*Luffa cylindrica* (L.) Roem (2*n* = 26), as the sponge gourd, is an important edible vegetable member of the Cucurbitaceae family in the Indian subcontinent. It is suitable for cultivation in the tropical and subtropical regions of the world during the summers and rainy seasons [4]. The edible part of this crop is tender fruits that can be consumed raw or cooked in various preparations [5]. Sponge gourd is also used in traditional medicine and reported to have nutraceutical benefits [6]. Indian tribes use sponge gourd to treat snake bites [7] and provide relief in labour pain and healing during childbirth [8]. In Korea, it is used to induce menstruation, maintain homeostasis, relieve phlegm and treat fever [9]. The plant extract has anti-inflammatory [10], anti-oxidant [11,12,13], anti-dryness [14] and anti-wrinkle [15] properties. It has recently been reported to have anti-obesity [16,17], anti-cancer [18,19] and suppression of SARS-CoV-2 symptoms qualities [20].

Aromatic germplasm of sponge gourd has been reported from Vietnam and Thailand [3]. The aromatic genotypes have also been reported in the cucumber and musk melon, a member of the same family. The aroma in cucumber is governed by a single recessive gene [21], and the principal odourant is (*E*,*Z*)-2,6 nonadienal. The main aromatic compound in rice, sorghum and soybean is 2-acetyl-1-pyrroline (2-AP) [1], which is also controlled by a single recessive gene. The main aroma compound in sponge gourd has also been identified as the 2-AP [3]. However, the aroma of mushrooms and certain other plants is linked with the mushroom alcohols, the eight carbon volatile compounds 1-octen-3-ol and 3-octanone [22].

During germplasm exploration in the Western Bihar, we collected several sponge gourd genotypes. These collections represent the natural population with random mating between the individuals and were maintained through selfing. During the first and second generation of selfing, we did not find any aromatic lines; however, during the third generation of selfing, an aromatic line was identified. This aromatic genotype was stabilised through selfing and used for the genetic studies and development of aromatic cultivars. The preliminary sensory evaluation by an expert panel confirmed aroma in this genotype and reported the presence of a Basmati rice-like aroma. Therefore, looking into the prospects of aromatic genotype, we conducted genetic analysis and deciphered the aromatic composition of sponge gourd fruits. This study is the first report on the collection and evaluation of aromatic sponge gourd from India.

## 2. Results

### 2.1. Characterisation of Parental Lines

**Figure 1:**
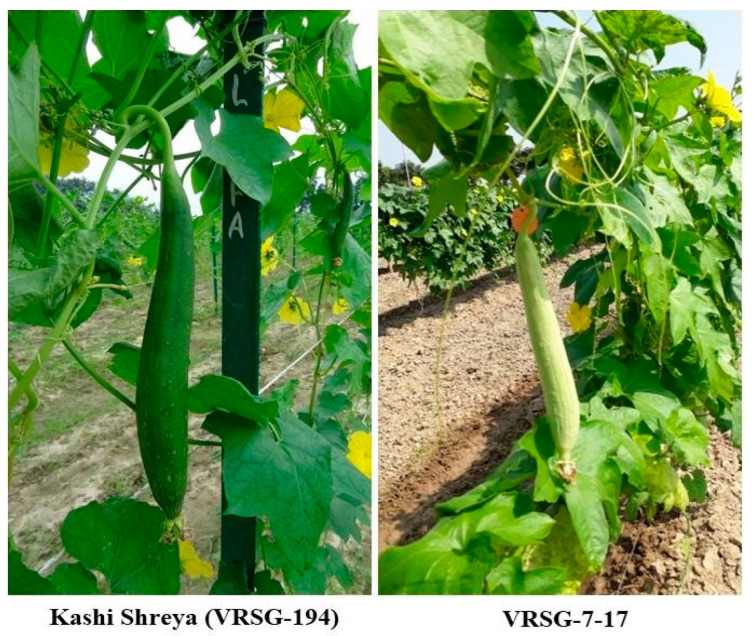
Morphological features of different parental lines of sponge gourd.

The parental lines were characterised for horticultural traits and aroma in different aerial plant parts. The aroma was present in the shoot, leaf and fruit tissues of the genotype VRSG-7-17. However, no aromatic fragrance was noticed in the genotype VRSG-194 grown under similar agronomic conditions. The fruit colour of VRSG-7-17 and VRSG-194 was light green and dark green, respectively (Figure 1).

As general characteristic features of the aromatic genotype VRSG-7-17, days recorded for Ist male and female flower appearance and fruit harvest were 43, 47 and 55.5, respectively, average fruit length was 27.46 cm, fruit diameter 3.35 cm, number of fruit per plant 3.6, vine length 6.28 m, fruit weight 156.5 g and yield per plant was 1.13 kg (Supplementary Table S1). The organoleptic test of cooked fruits of the parental lines by a group of four panel members confirmed a strong aroma smelling like the Basmati rice in the fruits of VRSG-7-17. The aroma of the cooked fruit of VRSG-7-17 kept at room temperature for 24 h with the lid of the Petri plate closed was retained intensely, suggesting strongly bound aromatic compounds with the fruit tissues.

### 2.2. Inheritance Studies of Aroma

The parental lines were crossed in *Kharif* 2019 to generate the F_1_s, and around 600 F_1_ seeds were obtained from the successfully crossed fruits. The F_1_s seeds were raised in the summer season of 2020 and a total of 50 plants were maintained in this generation through transplanting. All the F_1_s were found to be non-aromatic, suggesting the dominance of non-aromatic over the aromatic trait. The F_1_s were selfed and backcrossed with the parents to generate an ample quantity of F_2_ seeds. In the *Kharif* 2020 season, the 250 F_2_s, 100 BCP1, 100 BCP2 and 50 F_1_s along with parental lines were transplanted in the field and evaluated for aroma after fruiting. Fruits from individual plants were analysed for aroma. In the F_2_ generation, the progeny was segregated for the aroma, and 54 plants were found to be aromatic while 196 were non-aromatic. The Chi-square analysis of the segregation ratio for aromatic and non-aromatic lines suggested that the inheritance is governed by the Mendelian principle of segregation and the aroma is controlled by a single recessive gene. In the test cross population BCP1, a 1:1 segregation for aromatic (41) and non-aromatic plants (59) was observed (Table 1). All the plants in BCP2 and F_1_ were found to be non-aromatic. The results from the test cross and backcross progenies support the monogenic recessive inheritance of the aroma trait in sponge gourd.

### 2.3. Inheritance Studies of Fruit Colour

**Figure 2:**
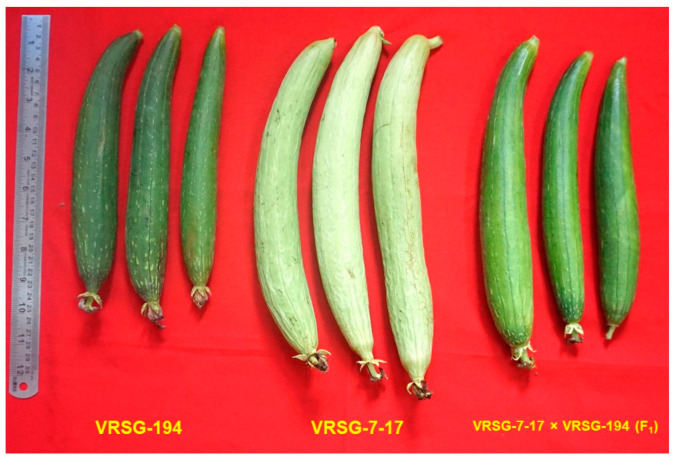
Fruits of parental lines VRSG-7-17, VRSG-194 and F_1_ (VRSG-7-17 × VRSG-194) showing light green colour, dark green colour and green colour, respectively.

The fruit colour of VRSG-7-17, VRSG-194 and F_1_s population was light green, dark green and moderate green, respectively (Figure 2). The inheritance analysis of fruit colour showed that this trait segregated into three classes. A total of 64 plants were with dark green fruits, 129 plants with green fruits and 57 plants having light green fruits. The Chi-square analysis showed that this ratio fits into the segregation ratio of 1:2:1 dark green, green and moderate green, respectively, indicating the existence of incomplete dominance for this trait. Further, the fruit colour and aroma were analysed together for possible linkage, and the plants were grouped into six classes namely non-aromatic dark green (NADG) (50), non-aromatic green (NAG) (99), non-aromatic light green (NALG) (47), aromatic dark green (ADG) (14), aromatic green (AG) (30) and aromatic light green (ALG) (10). The Chi-square analysis of these two traits segregated in the ratio of 3 (NADG): 6 (NAG): 3 (NALG): 1 (ADG): 2 (AG): 1 (ALG) (Table 2). This segregation ratio shows that these two traits are not linked and segregate independently of each other.

### 2.4. Organoleptic Test and Volatile Organic Compounds Analysis

For the analysis of volatile organic compounds, organoleptic scoring by team members gave a positive response suggesting the presence of Basmati rice-like aroma in ‘VRSG-7-17’ after physical smelling and cooking.

**Figure 3:**
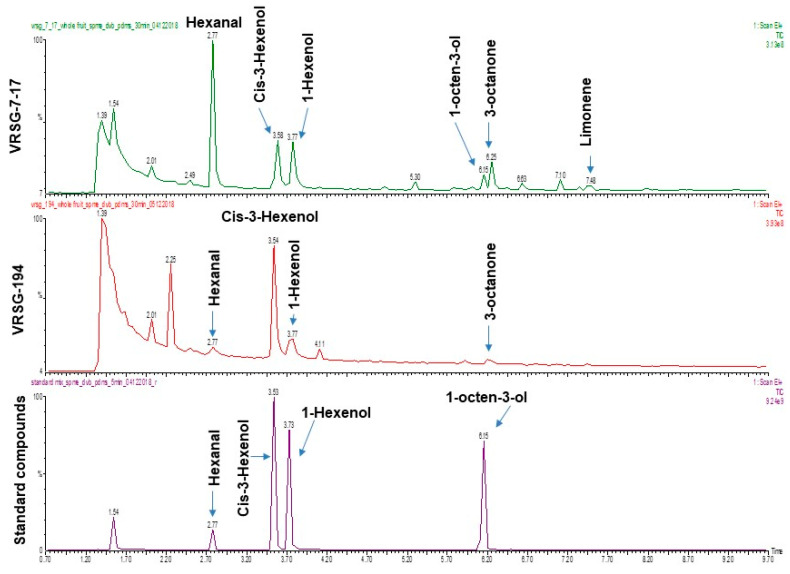
Typical GC–MS–SIM chromatograms of the extract obtained from the aromatic accession (VRSG-7-17) and VRSG-194 of sponge gourd along with the standard mix. The molecular formula of identified chemical compounds, e.g., (1) Hexanal (C_6_H_12_O); (2) *Cis*-3-Hexen-1-ol (C_6_H_12_O); (3) 1-Hexanol (C_6_H_14_O); (4) 1-Octen-3-ol (C_8_H_16_O); (5) 3-Octanone (C_8_H_16_O); (6) Limonene (C_10_H_16_O).

The SPME–GC/MS analysis revealed hexenal, 1-octen-3-ol and 3-octanone as major fatty acid derivatives followed by limonene as the only monoterpene hydrocarbon in ‘VRSG-7-17’ (Figure 3). On the contrary, *cis*-3-hexenol was found in ‘VRSG-194’. Other common volatiles identified using GC/MS were *cis*-3-hexenol and 1-hexenol. Therefore, the presence of hexanal, 1-octen-3-ol, 3-octanone and limonene in high proportion in ‘VRSG-7-17’ impart a Basmati-like pleasant aroma in this sponge gourd genotype.

## 3. Discussion

The present investigation was conducted to understand the inheritance and the aroma profile of an aromatic sponge gourd genotype, VRSG-7-17. The contrasting parents VRSG-194 and VRSG-7-17 were used to produce different crosses, and genetic studies were performed.

The aroma in sponge gourd is produced in different plant tissues such as leaves, stems and fruits. The aroma was also reported in the different aerial parts of rice plants [23]. The aromatic line VRSG-7-17 produced fewer fruits and yielded less fruits per plant than the non-aromatic genotype VRSG-194. The yield of Basmati rice has also been reported to be lower than that of non-aromatic genotypes. The energy required to produce the aroma and its associated secondary metabolites comes at the expense of yield accumulation [24,25].

### 3.1. Inheritance of Aroma

The inheritance of aroma was studied in crosses generated by crossing the sponge gourd genotype VRSG-7-17 and VRSG-194. The analysis of segregation in the F_2_ and backcross generations revealed that the inheritance of aroma traits in VRSG-7-17 is governed by a single recessive gene. The aroma has been reported in several crops, and the list has been comprehensively summarised by [1]. The inheritance of aroma has been reported to be controlled by recessive genes in rice [23], soybean [26] and sorghum [27]. In cucumber, inheritance is also governed by a single recessive gene [21]. The aroma in these crops is caused by a loss of function mutation in the functional genes, which causes them to be recessive in nature [1]. We also speculate that the aroma-regulating genes in the sponge gourd evolved through a loss of function mutation and then accumulation in the homozygous form. Because of the cross-pollinated nature of the crop, the recessive alleles remain hidden in the heterozygous plants, making it clear that aromatic genotypes are not found in high frequencies [28]. After being isolated from the natural habitat, such heterozygous plants may have resulted in the emergence of aromatic genotypes. The aroma may also be related to the negative fitness of the plants against the pest and diseases, therefore, a low frequency in the natural habitat. In the case of rice, aromatic genotypes were reported to be more susceptible to insect pests than non-aromatic genotypes [29]. Based on the recessive nature, we propose a symbol for the gene governing aroma as ‘*fgr*’ in sponge gourd.

### 3.2. Segregation of Aroma and Fruit Colour

Besides aroma, the parents involved in the crosses were also differing for the fruit colours. These two traits were studied concurrently for the possibility of linkage between fruit colour and aroma. Fruit colour inheritance studies revealed that the fruit colour is governed by a monogenic inheritance with incomplete dominance, i.e., in a segregation ratio of 1:2:1 for dark green, green and light green, respectively. The combined analysis of aroma and fruit colour showed that these two traits segregated independent of one another, with no evidence of a linkage. This could be due to the presence of genes governing these traits either on different chromosomes or very far apart on the same chromosome. The metabolic pathways that produce the fruit pigment and aroma may be regulated independently with no shared intermediates. The previous studies in sponge gourd show that the inheritance of fruit colour is controlled by a duplicate gene (15:1) with dark green being dominant over the light green colour [30]. However, the fruit’s skin colour is controlled by a single gene with incomplete dominance in the present study.

### 3.3. Volatiles and Organoleptic Taste

The genotype VRSG-7-17 was shown to have a rich aroma at the physical level of verification, which was attributed to volatile organic compounds. The presence of fatty acid derivatives such as hexanal, 1-octen-3-ol and limonene in ‘VRSG-7-17’ was revealed by GC/MS analysis of volatile compounds, which were retained in both fresh as well as in cooked fruits. The characteristics of the aroma molecules identified in this genotype are as follows the 1-octen-3-ol has been identified as an aliphatic alcohol commonly called “mushroom alcohol.” Hexanal produces fruity aroma, 3-octanone produces an aroma of lavender and limonene as the terpene compound produces a pleasant lemon grass-like odour [22,31,32,33]. Identification of two distinct classes of secondary metabolites in ‘VRSG-7-17’ suggested that two parallel biosynthetic pathways for fatty acids and terpenoids are active and responsible for the aroma. On the contrary, *Cis*-3-hexenol, a fatty acid pathway metabolite was found in ‘VRSG-194’. The presence of four different secondary plant metabolite classes as VOCs in ‘VRSG-7-17’ distinguishes it in terms of aromatic value, imparting a rich musky Basmati-like aroma. One accession of sponge gourd, ‘B29,’ was identified as having an aroma that was retained after cooking [28]. In melons, the volatile compound responsible for the musky aroma was reported to be 2-Acetyl-1-pyrroline (2-AP) [34]. The aroma in cucumber was also reported due to the presence of 2-AP [21]. In a recent report on aroma analysis in the sponge gourd and ridge gourd, Saensuk et al. [3] reported that the aroma in these crops was due to the 2-AP. The current study, however, demonstrates that the aroma in the genotype ‘VRSG-7-17’ is due to the presence of compounds other than 2-AP. These volatiles have been identified in mushrooms, camembert cheese, blue cheese, raspberries, orange juice, elder flower and other plants [22,32,33]. Furthermore, in nature, 1-octen-3-ol serves as a signalling molecule in plant cellular responses, plant–herbivore interactions and plant–plant interactions. For example, in *Arabidopsis*, 1-octen-3-ol induces expression of defence genes that are normally upregulated by wounding or ethylene/jasmonic acid signalling. In addition, treatment with 1-octen-3-ol inhibits the expansion of necrotic lesions on *Arabidopsis* leaves [32]. The presence of 2-AP, on the other hand, has been associated with the susceptibility to biotic and abiotic stresses [24].

### 3.4. Breeding Strategies for Aroma in Sponge Gourd

Aroma is governed in a simple Mendelian fashion with recessive inheritance, so the pedigree selection could be the most appropriate breeding strategy to generate variation, followed by the selection of desired plant types along with the aroma. Backcross breeding can also be utilised to transfer the aroma trait into promising genotypes or cultivars. However, the selection of recessive aroma traits must be carried out in the segregating generation, which will prolong the breeding cycle. Until linked markers for this trait are developed, selection of segregants with proper aroma scoring will be critical in the breeding programme. The utilisation of this trait in hybrid breeding for harnessing the heterosis for yield and other traits can be achieved by transferring this trait into the two parental inbred lines of the particular hybrid.

## 4. Materials and Methods

### 4.1. Plant Materials

The plant materials consisted of aromatic and non-aromatic genotypes of *Luffa cylindrica*. The crosses were made between the aromatic (VRSG-7-17) and non-aromatic (VRSG-194) lines differing in fruit colour. Tender fruits from these F_1_s were rated for the aroma to determine if the aroma was a dominant trait. Segregation analysis of F_2_s based on aroma rating of tender fruits from individual F_2_ plants was carried out. The genotype VRSG-7-17 was found to produce a sharp and easily identifiable aroma characteristic of scented Basmati rice. In *Kharif* 2020, F_1_s, F_2_, BCP1 (F_1_ × VRSG-7-17) and BCP2 (F_1_ × VRSG-194) progenies from all the crosses were grown in the field. The seedling of parents, F_1_s, F_2_, BCP1 and BCP2 from each cross were grown in the 20 cells portrays (37 cm × 30 cm × 7.5 cm) and transplanted in the main field, where they were grown under standard management practices.

### 4.2. Making of Crosses

The crossing is relatively easy in *Luffa cylindrica* because the plants produce monoecious flowers on different nodes, requiring no emasculation of the flower and producing a large number of seeds from a single fruit. On the prior evening of making crosses, the female flower buds were covered with butter paper so that outcrossing can be prevented. The next morning, the fully open male flower of the VRSG-194 was selected and the anther portion was touched on the stigma of female flowers of VRSG-7-17. Following pollination, the female flowers were again covered with the butter paper for the next five days. The fruits from the crossed and selfed plant were harvested when the skin of the fruits began to dry and turn brown. A single successfully pollinated fruit can yield up to 150 seeds. The plants selected for the crossing had different fruit colours, i.e., light green (VRSG-7-17) and dark green (VRSG-194). The appearance of green fruits in the crosses (F_1_) involving light-green-fruited genotypes as female and dark-green-fruited genotypes as male indicated the successful crossing, thus acting as a morphological marker for the selection of true crosses.

### 4.3. Fruit Aroma Test

To test the presence of aroma, 3 g of fresh sample from the tender fruit was taken. The skin of the fruit was removed with the help of vegetable peeler to reduce the strong chlorophyll smell in the test sample. The fruit pulp was cut into tiny pieces and put into disposable Petri dishes. We followed the same protocol that was used for the aroma testing in rice [23]. To the 3 g of fresh fruit pulp, 10 mL of 1.7% KOH was added. The Petri plates were immediately covered and left at room temp for 15 min prior to opening one by one for the aroma test. Each plate was smelt and scored for the presence and absence of aroma by a panel of 4 members who gave ratings of smell, as per the previously reported method of Sagar et al. [35,36]. Similarly, cooked samples of the scented and non-scented fruits were also checked for aromatic smell, as per the procedure described above.

### 4.4. Extraction of Volatile Organic Compounds

For the analysis of volatile organic compounds (VOCs), the aromatic genotype ‘VRSG-7-17’ was raised in earthen pots containing 6.5 kg of field soil per pot under glasshouse conditions. Kashi Shreya (VRSG-194) served as control (non-scented). Analysis for VOCs was carried out with 15 plants in triplicate. The use of chemicals and fertilizers was avoided throughout the crop raising.

The solid-phase microextraction (SPME) technique was used to extract VOCs from fruits of the plants. Fruits were cut into small pieces and placed in a sealed flask in a glass chamber to attain equilibrium. The activated fibre comprised divinylbenzene/polydimethylsiloxane-coated phase of 65-micrometer thickness, fitted in a manual SPME holder 24 GA (Supelco, Bellefonte, PA, USA). The activated fibre was exposed to the headspace region of the glass chamber containing the sponge gourd samples individually for 15 min. The fibre was then desorbed into the heated GC–MS injector (PSS type). In between the two consecutive runs, a blank run was also performed using PDMS/DVB fibre.

### 4.5. Gas Chromatography (GC), Chiral GC and GC–Mass Spectrometry Analysis

The GC and GC–MS analyses were performed as per the method reported earlier [37]. Relative retention indices were calculated by injecting a homologous series of *n*-alkanes (C_6_-C_28_ hydrocarbons, Polyscience Corp, Niles, IL, USA). In addition, chiral standards (Aldrich (St. Louis, MO, USA) and Fluka (St. Gallen, Switzerland) were injected using the same oven programme. Compound identification was achieved by comparing two MS libraries (Turbo Mass NIST 2011 version 2.3.0 and Wiley registry of mass spectral data 9th edition) and a reference guide on mass spectral data [38].

### 4.6. Recording of the Horticultural Traits

The morphological traits contrasting between the parents were also recorded for F_1_s, F_2_s, BCP1 and BCP2, as per the descriptors data set provided for the *Luffa cylindrica*. The RHS colour chart was used to classify genotypic differences for colour variation.

### 4.7. Statistical Analysis

The data were analysed for the differences in the mean using Tukey’s test. The Chi-square (χ^2^) value of the aroma and fruit colour data obtained from the segregating population of the crosses were computed following the procedure described by Gomez and Gomez [39].

## 5. Conclusions

The translation of aroma into the sponge gourd breeding programme can only be accomplished with a thorough understanding of the genetic control of this trait. The analysis of inheritance of this trait culminated in the finding that this trait is governed by a single recessive gene in the sponge gourd genotype VRSG-7-17. According to the SPME and GC–MS analysis, the four compounds hexanal, 1-octen-3-ol, 3-octanone and limonene were the dominant volatiles present in the aromatic genotype, giving it a distinct aroma in the aerial parts.

## Figures and Tables

**Table 1:** Observed segregation ratio and the Chi-square (χ^2^) value for the goodness-of-fit to monogenic model for F_2_ and test cross population derived from a cross between aromatic and non-aromatic *Luffa* lines.

Generation	Total No. of Plants	Non-aromatic	Aromatic	Expected	Calculated χ^2^ Value	*p* Value (0.05)
P1(VRSG-194) Non-aromatic	10					
P2(VRSG-7-17) Aromatic	10					
F_1_	50	50	-		-	-
F_2_	250	196	54	3:1	1.54	0.214
BCP1	100	59	41	1:1	3.24	0.071
BCP2	100	100	-		-	-

**Table 2:** Observed segregation ratio and the Chi-square (χ^2^) value for the goodness-of-fit to monogenic model (fruit colour) and digenic model (aroma and fruit colour) in an F_2_ population derived from a cross between aromatic, light green and non-aromatic, dark green Luffa lines.

Generation	Total Plants (No.)	Dark Green (DG)	Green			Light Green (LG)		Expected	Calculated χ^2^ Value	*p* Value (0.05)
P1(VRSG194)	10	DG						1:0:0		
P2(VRSG-7-17)	10					LG		0:0:1		
F_1_	50		50					0:1:0	-	-
F_2_	250	64	129			57		1:2:1	0.64	0.72
**Generation**	**Total plants (No.)**	**NADG**	**NAG**	**NALG**	**ADG**	**AG**	**ALG**			
F_2_	250	50	99	47	14	30	10	3:6:3:1:2:1	3.85	0.57

Non-aromatic dark green (NADG); non-aromatic green (NAG); non-aromatic light green (NALG); aromatic dark green (ADG); aromatic green (AG); aromatic light green (ALG).

## Data Availability

Data will be available through a request to the corresponding author.

## Supplementary Materials

The following supporting information can be downloaded at: https://www.mdpi.com/article/10.3390/plants11212881/s1, Table S1: Agronomic features of parental lines and hybrids used in the aroma analysis based on an average of two years of observation.
