# Supplementary material for: Volatile Compounds Governed by Single Recessive Gene Impart Aroma in Sponge Gourd (Luffa cylindrica L. Roem)
Source: Plants (Basel). 2022 Oct 28;11(21):2881. doi: 10.3390/plants11212881 (PMC9656515; doi:10.3390/plants11212881)
Supplement: Supplementary file 1 [file plants-11-02881-s001.zip › plants-1946599-supplementary.pdf]

Supplementary Table S1. Agronomic features of parental lines and hybrids used in the aroma analysis based on an average of two years of observation. Different letter superscripts are significantly different.

**Particulars**	**VRSG7-17**	**Kashi Shreya (VRSG-194)**	**Kashi Shreya (VRSG-194) × VRSG-7-17**
Days to 1st male flower appearance (Days)	43.0^{a}	37.5^{b}	40.5^{c}
Days to 1st female flower appearance (Days)	47.0^{a}	38.0^{b}	43.5^{c}
Days to first fruit harvest (days)	55.5^{a}	43.5^{b}	50.5^{c}
Fruit length (cm)	27.46^{a}	26.78^{a}	31.17^{b}
Fruit diameter (cm)	3.35^{a}	3.55^{b}	3.8^{c}
Number of fruits per plant (No.)	3.6^{a}	8.6^{b}	7.7^{b}
Fruit weight (g)	156.5^{a}	149.0^{b}	192.0^{c}
Vine length (m)	6.28^{a}	5.81^{b}	6.29^{a}
Yield/Plant (Kg)	1.13^{a}	1.78^{b}	1.47^{c}
Fruit colour	Light green (143C)	Dark green (137B)	Green (140B)
Aroma	Aromatic	Non-aromatic	Non-aromatic
